# Accounting for detection unveils the intricacy of wild boar and rabbit co-occurrence patterns in a Mediterranean landscape

**DOI:** 10.1038/s41598-020-63492-9

**Published:** 2020-04-20

**Authors:** Ana Luísa Barros, Gonçalo Curveira-Santos, Tiago André Marques, Margarida Santos-Reis

**Affiliations:** 1 cE3c – Centre for Ecology, Evolution and Environmental Changes, Faculdade de Ciências da Universidade de Lisboa, Ed. C2, Campo Grande, 1749-016 Lisbon, Portugal; 2 Centre for Research into Ecological and Environmental Modelling, The Observatory, University of St Andrews, St Andrews, KY16 9LZ Scotland; 3 Centro de Estatística e Aplicações, Departamento de Biologia Ambiental, Faculdade de Ciências, Universidade de Lisboa, 1749-016 Lisbon, Portugal

**Keywords:** Conservation biology, Ecological modelling

## Abstract

The patterns of species co-occurrence have long served as a primary approach to explore concepts of interspecific interaction. However, the interpretation of such patterns is difficult as they can result from several complex ecological processes, in a scale-dependent manner. Here, we aim to investigate the co-occurrence pattern between European rabbit and wild boar in an estate in Central Portugal, using two-species occupancy modelling. With this framework, we tested species interaction for occupancy and detection, but also the interdependencies between both parameters. According to our results, the wild boar and European rabbit occurred independently in the study area. However, model averaging of the detection parameters revealed a potential positive effect of wild boar’s presence on rabbit’s detection probability. Upon further analysis of the parameter interdependencies, our results suggested that failing to account for a positive effect on rabbit’s detection could lead to potentially biased interpretations of the co-occurrence pattern. Our study, in spite of preliminary, highlights the need to understand these different pathways of species interaction to avoid erroneous inferences.

## Introduction

Understanding the patterns of species co-occurrence is of fundamental interest for many fields of ecological research. Such knowledge underpins much of our understanding of concepts like community assembly^1^, competitive exclusion^2^, niche partitioning^3^ and predator–prey dynamics^4^. In particular, interspecific dependencies in species co-occurrence have served for long as a primary approach to explore concepts related to interspecific interactions^2,5,6^. The way in which a species occurrence probability is conditioned by the presence or absence of a potential competitor, can serve as a non-mechanistic proxy^7^ for interference competition or facilitation. However, the interpretation of co-occurrence patterns is inherently difficult. Such patterns can result from complex ecological processes acting in tandem, in a scale-dependent manner^8,9^, or from shared/distinct habitat requirements. For instance, similar habitat preferences could lead to overlapping distributions at a broader scale but, at a patch level, risk/avoidance behaviour of the subordinate species^9^ can be interpreted as spatial displacement. Therefore, at a fine scale, effects of interspecific interaction tend to be more subtle and difficult to demonstrate, as they can manifest in complex ways^2^ (e.g. activity patterns, space use, species abundance). Given the ecological and conservation relevance^4,6,10^ of understanding species co-occurrence patterns, it is important that the complexity underlying these interactions is acknowledged and dully accounted for.

Observational studies to discuss hypotheses on interspecific interactions can be useful when controlled experiments are logistically unfeasible^11^. Two-species occupancy modelling^12^ has emerged as a tool to investigate hierarchical interactions between co-occurring species^13,14^. Importantly, this method allows to conditionally model the probability of occupancy and detection of a subordinate species upon the presence and/or detection of the dominant species^12^. Furthermore, the possibility to include habitat variables^15^ allows to interpret such patterns, while accounting for habitat preferences. Past studies provided evidence in support of interspecific dependencies for occupancy and detection probabilities, attributing this to different ecological processes^4,10,16^. A multi-level approach is important because a fine scale avoidance behaviour by the subordinate species could reduce detectability and consequently lead to an overestimation of the spatial exclusion pattern. This is also true if fine scale habitat modifications by the dominant species increase detection of the subordinate species^8^, which would skew inferences of species co-occurrence. However, few studies consider such interdependencies between detection and occupancy parameters^17^. Such can be important to unveil new interaction pathways, that would otherwise be masked by unmodelled factors, and/or provide unbiased estimates of the co-occurrence parameter of interest.

European rabbit (*Oryctolagus cuniculus*) and wild boar (*Sus scrofa*) often co-occur with contrasting population densities and a few previous studies^18–20^ investigated a potential interspecific interaction. For the past decades, populations of wild ungulates, such as the wild boar, have been growing all over Europe^21^ and particularly in the Iberian Peninsula^22^. As a generalist omnivore, the wild boar uses soil rooting to forage. It also wallows in the mud to cool down and remove ectoparasites^23^. These behaviours alter soil composition and pH levels, decomposition processes^24^ and can reduce nearly 80% of the herbaceous cover^25^, reducing plant diversity and regeneration. These effects on micro-habitat structure may cause the European rabbit to alter the intensity of fine scale space use when wild boar is present, potentially avoiding rooted areas and searching for cover^26^. Therefore, although co-occurring, signs of presence would be less evident and therefore less detectable. Furthermore, rooting increases soil compaction and nitrogen availability, thus favouring nitrophilous species and reducing leguminous availability^27^. This affects the rabbit’s breeding success since leguminous are essential during this period, and these also constitute an important protein source for wild boar. Moreover, in-depth rooting compromises underground refuge stability (e.g. burrows) for rabbits and there are even some reports of direct predation^28^. Therefore, direct competition may cause European rabbit to abandon sites less suitable in terms of food and shelter due to wild boar’s presence, creating a pattern of spatial displacement.

This potential interspecific competition could further impact already depleted European rabbit populations. These have been declining since the 20^th^ century^29^, although the species can be locally perceived as a pest within its native range^30^. Furthermore, it is native to the Iberian Peninsula^31^ and considered a keystone species^32^. The European rabbit is threaten mainly due to the outbreak of two viral diseases: myxomatosis and viral haemorrhagic disease^33^. These exacerbated the on-going decline due to habitat loss and fragmentation^29,34^, while other relevant threats include predation pressure^35^, inadequate hunting practices^36^ and interspecific competition^20^. More recently, a new variant of the rabbit haemorrhagic disease virus (RHDV2) continues to devastate populations, especially those in low density^37^. Consequently, the European rabbit is classified as “Nearly Threatened” in Portugal^38^ and “Vulnerable” in Spain^39^. However, a recent re-assessment from the IUCN has classified this species as “Endangered”^40^, as the population continues to decline. Therefore, wild boar could hamper the recovery of depleted European rabbit populations. Even more so, given intensive management tends to increase wild boar’s numbers as this is a relevant big game species in the Iberian Peninsula^41^.

Here we aim to investigate the co-occurrence patterns between the European rabbit and the wild boar in an agroforestry estate in Central Portugal, to elucidate potential interaction pathways. Within a two-species occupancy modelling framework, we specifically evaluated standing expectations that (i) wild boar presence had a negative influence on rabbit’s occupancy, (ii) and detection probabilities; and (iii) interdependencies between conditional occupancy and detection probabilities influenced the estimation of co-occurrence patterns. We also included habitat covariates to try to disentangle the effect of wild boar’s presence from shared habitat preferences. Testing such hypotheses will further contribute to comprehend the complexity of species co-occurrence patterns and their implications for future research.

## Materials and methods

### Study area

**Figure 1:**
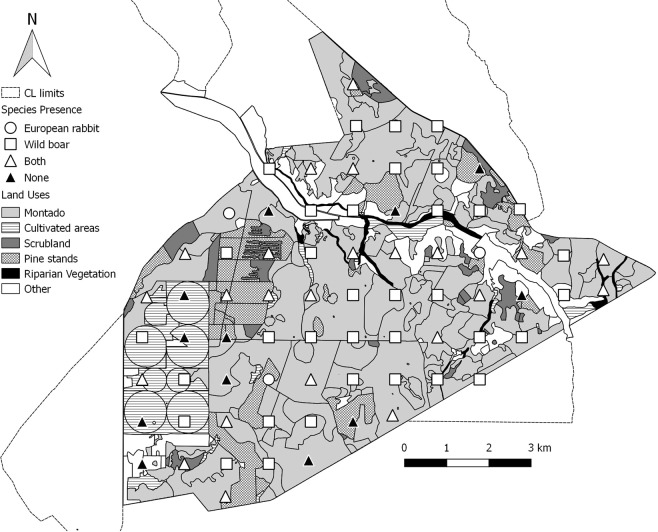
Location of the study area in Portugal and sampling design in Charneca, Companhia das Lezírias (CL) (map created in QGIS 3.12.0, http://qgis.osgeo.org). Depiction of the main land-cover types and the 73 sampling points (sites), 1 km apart, with species- and site-specific detection patterns (open circles for European rabbit, open squares for wild boar, open triangles for both species and closed triangles for neither).

The study site was the “Charneca”, NE section (100 km^2^) of Companhia das Lezírias S.A., the largest agroforestry farmstead in Portugal, with nearly 180 km^2^. This area was primarily used for silviculture and pastoral practices. Over the years, different management options shaped a complex landscape, where cork oak Montado was the primary land-cover (~66 km^2^). The Montado occurred in pure or mixed patches and with variable composition and density of understory, depending on grazing pressures and/or shrub clearance activities. This landscape was interspersed by maritime pine stands, scrublands, olive groves, rice fields and irrigation plots^42^ (Fig. 1).

The study site was also explored as a hunting estate where game species such as the European rabbit and wild boar were targeted. In Companhia das Lezírias’ Forest Management reports^43^, rabbit’s local population has been recognised as declining. Since 2008 several conservation measures have been implemented (e.g. scrubland management, food and water supply network, population monitoring) and hunting ceased in 2013. Contrasting, the wild boar has been growing steadily in population numbers and was widespread. As a consequence, the population was controlled through intensive hunting practices^43^ and during our study, four hunting events occurred (16 December 2015, 20 January 2016, 10 February 2016, 24 February 2016).

### Field sampling protocol to assess species presence

We surveyed rabbit and wild boar signs of presence between December 2015 and March 2016. We used a regular sampling strategy by dividing the area into 1 × 1 km plots and prospecting a 25 meters radius buffer around the centre of each plot (i.e. sites, N = 73; Fig. 1). Presence was confirmed by signs such as pellets, latrines and burrows in the case of European rabbit, and droppings, footprints and rooted area in the case of wild boar. Each buffer was prospected by the same two observers, who actively searched for these signs of presence. A species was considered present at a site if at least one of these signs was detected.

We conducted three sampling occasions and during each occasion, all sites (n = 73) were surveyed once, over a period of one to one and a half weeks ($\bar{X}$ = 9 days, SE = 1.528), depending on logistic constraints. Sampling occasions were spaced by a period of two to three weeks. All signs were registered so that in the next sampling occasion we could exclude highly persistent signs such as rabbit burrows, ensuring temporal independence between occasions. Rabbit and wild boar detections were coded as 1 for detected and 0 for non-detected, which allowed us to build site- and species-specific detection histories. For example, the detection history ${X}_{l}^{A}$ = 001, means the location i was surveyed on three occasions, with species A being detected only in the last occasion. Overall, we had four sites with missing observations in the last two sampling occasions.

### Occupancy modelling

Occupancy models make use of spatial-temporal replicated data to generate a likelihood based estimate using probabilistic arguments that account for false absences^44,45^. Site- and survey-level covariates can be incorporated when estimating both detection and occupancy parameters via a logit link^45^. Two species occupancy modelling^12,44^ extends this approach allowing the probability of occupancy and detection of a subordinate species to be modelled as a function of the occupancy and detection status of the dominant species. A species interaction factor (SIF) is calculated as a ratio of how likely the two species are to co-occur compared to what would be expected under a hypothesis of independent occupancy^15^. A value <1 suggests avoidance, while values >1 indicate co-occurrence. This parameterization assumes: *i*) sites are closed to changes in the occupancy status of the species, *ii*) sites and occasions are, respectively, spatially and temporally independent, and *iii*) there is no un-modelled heterogeneity in both detection and occupancy. Here, we relaxed these assumptions by assuming that, during our sampling period: rabbit populations were stable, as the survey would have ended before the reproductive season^46^, and wild boar’s occupancy was interpreted as probability of site use^12^, given it is a highly mobile species with large home ranges and likely random movement patterns^47^ within the estate (assumption *i*,); furthermore, the time interval between sampling occasions (>2 weeks) mitigated the temporal dependence (assumption *ii*); and the inclusion of detection and occupancy covariates achieved a compromise between model complexity and interpretability (assumption *iii*).

We used a two-stage modelling approach^14^: firstly, we used single-species occupancy models to identify relevant covariates for both species and to avoid overparameterization in the next stage^15^; secondly, we used two-species models, to assess interspecific interactions. For single-species models, we first tested the effect of the covariates on species detection (p) while keeping occupancy (ψ) constant; then carried the covariate from the best-fitting model and built a second set of candidate models explaining species occupancy probabilities^3^. For detectability we considered habitat features within the surveyed buffers, but also the rabbit’s marking behaviour. Since in our study area European rabbit occurs in low density, large latrines are uncommon^48^ and scattered pellets were more often used to ascertain species presence. We considered vegetation influenced the detection of such signs and therefore measured the following variables: shrub (S), herbaceous (H), and litter cover (L), shrub (SH) and herbaceous height (HH), and percentage of bare ground (B). For each site (N = 73), the same two observers visually estimated an average value of cover percentage and height, during the first sampling occasion. We also used Julian day (JD) as a sampling covariate as the interval between sampling occasions could have influenced species detection.

For occupancy, we selected covariates related to habitat, food availability and disturbance. The wild boar has been described as a generalist species, with preference for forested areas with dense shrub cover and high food availability (e.g. acorns)^49^. Studies on European rabbit established the importance of landscape mosaics of scrublands and pastures^50^. However, the presence of livestock poses a threat to rabbit populations, and ultimately to wild boar, as high grazing pressure leads to shrub clearance and landscape simplification^51^. The covariates tested are described in Table 1. These were extracted for a 100 meters radius buffer around each site (according, approximately, to rabbit’s largest home-range size in Doñana, Spain^52^) in software Quantum GIS (QGIS Development Team, 2016), using a GIS database available for the study area^3^. We mostly tested univariate models, but also a few with covariate combinations representing specific ecological hypothesis (e.g. food and habitat: Mont+Sparse+Cult). We assessed covariate correlation and to avoid multicollinearity excluded the less ecologically meaningful covariate when the coefficient surpassed 0.7^53^. Herbaceous cover and shrub height covariates were excluded from the analysis (see Supplementary Table S1 online). Also, the dense scrubland covariate led to convergence errors when modelling wild boar’s occupancy and was thus removed for this species. Prior to the analysis, all covariates were standardized to z-scores.

**Table 1:** Covariates selected to model European rabbit (ER) and wild boar (WB) occupancy probability (ψ) in Charneca (CL), according to species’ ecological requirements. For each covariate an abbreviation code and brief description are presented.

Covariate	Code	Description	Species
Dense scrubland	Dense	Undisturbed forest and scrubland patches with > 60% understory cover, dominated by *Ulex* sp., *Cistus ladanifer* and *Cistus monspeliensis*.	ER
Sparse scrubland	Sparse	Semi-disturbed forest and scrubland patches, with understory cover between 30–60%, and moderate grazing pressures.	ER/WB
Absent scrubland	Absent	Highly disturbed forest patches with <30% understory cover due to intense grazing pressure and/or shrub clearance activities.	ER/WB
Montado	Mont	Agrosilvopastoral system dominated by *Quercus suber*, with varied understory densities depending on grazing pressures and shrub clearance activities. Ground mostly covered by natural/permanent pastures.	ER/WB
Pine stands	Pine	Dominated by *Pinus pinaster*. Stands of varying age, with sparse understory cover.	ER/WB
Landscape heterogeneity	Div	Patch diversity in a 100 meters radius buffer, measured by the Shannon diversity index ( $H=-\mathop{\sum }\limits_{i=1}^{m}Pi\ast lnPi$)	ER/WB
Riparian vegetation	Rip	Dense vegetation adjacent to waterlines, with mixed composition of *Salix* alba, *Fraxinus angustifolia*, *Alnus glutinosa*, *Crataegus monogyna* and *Rubus fruticosus*. Measured as the distance, in meters, from the buffer’s centroid.	WB
Cultivated areas	Cult	Area dedicated to agricultural practices, composed of olive groves, irrigated areas for forage production and fallow land. Measured as the distance, in meters, from the buffer’s centroid.	ER/WB
Artificial feeding	Art	Points of artificial food and water supply for European rabbit. Measured as the distance, in meters, from the buffer’s centroid.	ER
Cattle disturbance	Cattle	Grazing pressure index given by $\,LSU/(ha\times n)$, where LSU is the number of livestock units in a plot of a given area (ha) during a certain number of days (n). Weighted average as a function of the grazing plot area within the 100 meters radius buffer.	ER/WB

In the second stage, we tested for species interaction using the best supported covariates previously identified and following the parameterization of Richmond *et al*. (2010) (see Table 2 for parameter abbreviations). Wild boar (“WB” indicates present; “wb” absent) was considered the dominant species and European rabbit (ER) the subordinate species. To test the hypotheses outlined in the Introduction we built four types of models:

(i) both occupancy and detection probabilities are unconditional (ΨWB ΨER pWB pER);
(ii) occupancy is unconditional but detection is conditional (ΨWB ΨER pWB pER rER/WB);
(iii) occupancy is conditional but detection is unconditional (ΨWB ΨER/WB ΨER/wb pWB pER);
(iv) both occupancy and detection are conditional (ΨWB ΨER/WB ΨER/wb pWB pER rER/WB).

**Table 2:** Single-season two-species occupancy model parameters and definition in accordance with the parameterization of Richmond *et al*., 2010. “WB” stands for wild boar present, “wb” for wild boar absent and “ER” for European rabbit.

Parameter	Definition
ψWB	Species WB occupancy probability
ψER/WB	Species ER occupancy probability with species WB present
ψER/wb	Species ER occupancy probability with species WB absent
pWB	Species WB detection probability with species ER absent
pER	Species ER detection probability with species WB absent
rWB	Species WB detection probability with species ER present
rER/WB	Species ER detection probability with both species present and species WB detected
rER/wb	Species ER detection probability with both species present and species WB not detected

### Model selection

Prior to model ranking we determined the goodness-of-fit of each species global model, using the Pearson chi-square statistic (1000 parametric bootstrap samples). Following the methodology described in MacKenzie and Bailey (2004)^54^, we used the Akaike Information Criterion corrected for small sample size (AICc) for model ranking. This approach assumes that, within the candidate set, models with ΔAICc ≤ 2 comparatively to the best model are strongly supported and their covariates are good predictors of the dependent variable. However, the use of an apparently arbitrary cut-off value has been previously criticized, and other values of ΔAICc of 4, 6 or even 8 may also include relevant models^55^. Thus, for the selection of single-species models we maintained a stricter rule of ΔAICc ≤ 2 to avoid overparameterization in the next stage. Then, for the two-species model analysis we resorted to the Akaike’s weight (AICw), which indicates the weight of the evidence in favour of a certain model being the most parsimonious in the set. According to Burnham and Anderson (2002)^56^, unless a single model has a AICw >0.9, then other models are likely to be relevant and therefore should also be considered for inference. In the two-species model set, we compared AICw between models with conditional occupancy and/or detection probabilities to compare the evidences supporting our hypothesis.

We used model averaging across the two-species model set to obtain parameter estimates of occupancy and detection probabilities. This also allowed us to compare the different hypothesis, since: if ψER/WB < ψER/wb, rabbit’s occupancy probability is lower at sites where the wild boar is present, which would indicate spatial displacement (SIF < 1). If p^ER^ > r^ER^, rabbit’s detection probability is higher when wild boar is absent, and therefore detection is negatively influenced by wild boar’s presence. The covariate effect was also ascertained by model averaging. If a covariate was not included in a given model the value was set to zero, and we calculated the weighted average using the respective AICw^57^. For the standard error (SE) we used the formula from Anderson (2008)^58^ and estimated 90% unconditional c onfidence intervals (CI). We considered a well-supported effect when this interval did not overlap zero. Single-species single-season modelling was done in R statistical software (version 3.4.4) using the “unmarked” package^59^, and two-species single-season modelling was done in software PRESENCE 11.3^60^.

## Results

Overall, European rabbit’s naïve occupancy (i.e. proportion of sites where species was detected) was 0.36 (26 of the 73 sites), which contrasts with the wild boar with 0.80 naïve occupancy (58 out of 73 sites; Fig. 1). European rabbit and wild boar were detected together in 32% of sites, meaning rabbit was detected alone in only three of the 26 sites it was detected at.

Model averaging of the top-ranking single-species models indicated a well-supported negative effect of the proportion of pine stands on wild boar’s occupancy probability and a negative effect of the distance to cultivated areas on European rabbit’s occupancy (see Supplementary Tables S2 and S3). Also, litter cover had a negative effect on wild boar’s detection probability and shrub cover had a positive effect on rabbit’s detection, both well-supported (see Supplementary Tables S3 and S4).

To build the single season two-species candidate model set we combined the four types of models described in the “Occupancy modelling” sub-section, with these four well-supported detection and occupancy covariates. This model set comprised 16 models (see Supplementary Table S5), however, for final inference, we maintained only the top better fitted 12 models (Table 3). A few of the least supported models had convergence errors and therefore we discarded them, and all inferences were done for this subset that accounts for 90% of the explained variance. From this set, models that support some type of interaction, either for detection, occupancy or both, had a cumulative AICw of 0.569.

**Table 3:** Model ranking of single season two-species occupancy models testing the different scenarios for species interaction and according to the hypothesis presented.

Hypothesis	Model	K	AIC	ΔAIC	AICw	SIF
Ψ(un), p(un)	ψWB(Pine) ψER(Cult) pWB(L) pER(S)	8	479.82	0	0.183	1
Ψ(cond), p(un)	ψWB(.) ψER/WB(Cult) ψER/wb(.) pWB(L) pER(S)	8	480.81	0.99	0.112	>1
Ψ(un), p(un)	ψWB(Pine) ψER(.) pWB(L) pER(S)	7	480.97	1.15	0.103	1
Ψ(cond), p(un)	ψWB(Pine) ψER/WB(Cult) ψER/wb(.) pWB(L) pER(S)	9	480.98	1.16	0.103	>1
Ψ(un), p(un)	ψWB(.) ψER(Cult) pWB(L) pER(S)	7	481.16	1.34	0.094	1
Ψ(un), p(cond)	ψWB(Pine) ψER(Cult) pWB(L) pER(S) rER/WB=rER/wb(.)	9	481.67	1.85	0.073	1
Ψ(cond), p(cond)	ψWB(Pine) ψER/WB(Cult) ψER/wb(.) pWB(L) pER(S) rER/WB=rER/wb(.)	10	481.69	1.87	0.072	<1
Ψ(un), p(cond)	ΨWB(Pine) ΨER(.) ρWB(L) ρER(S) rER/WB=rER/wb(.)	8	482.12	2.3	0.058	1
Ψ(cond), p(un)	ΨWB(Pine) ΨER/WB(.) ΨER/wb(.) ρWB(L) ρER(S)	8	482.16	2.34	0.057	>1
Ψ(un), p(un)	ΨWB(.) ΨER(.) ρWB(L) ρER(S)	6	482.31	2.49	0.053	1
Ψ(cond), p(un)	ΨWB(.) ΨER/WB(.) ΨER/wb(.) ρWB(L) ρER(S)	7	482.42	2.6	0.05	>1
Ψ(cond), p(cond)	ΨWB(Pine) ΨER/WB(.) ΨER/wb(.) ρWB(L) ρER(S) rER/WB=rER/wb(.)	9	482.66	2.84	0.044	<1

The models with convergence errors were discarded maintaining the sub-set that accounted for a cumulative AICw of 0.9. Model parameters are described in Table 2 and covariate abbreviations in Table 1. “WB” stands for wild boar, “ER” for European rabbit, “un” for unconditional and “cond” for conditional.

**Figure 2:**
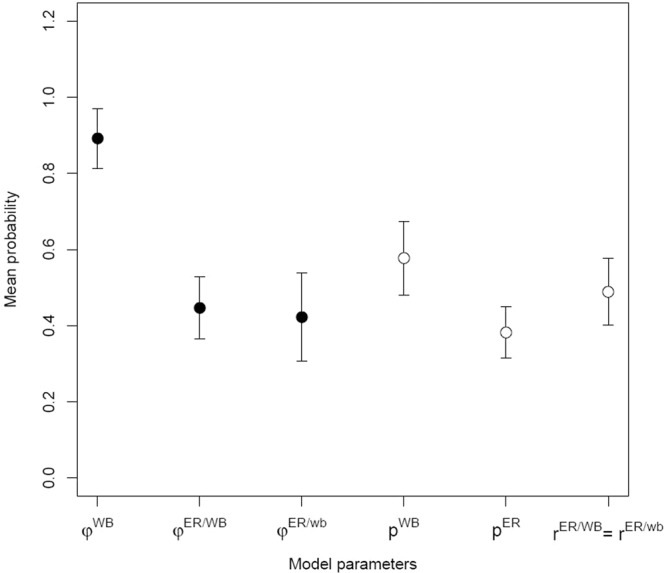
Model average of parameter estimates across the single-season two-species occupancy model set for European rabbit and wild boar, and respective standard error. Occupancy probability estimates (ψ) are represented by closed circles and detection probability estimates by open circles (p). Model parameters are described in Table 2.

Our first hypothesis of a negative effect on European rabbit’s occupancy by wild boar’s presence was not strongly supported given independence models for occupancy probability (ψ^ER/WB^ = ψ^ER/wb^; AICw = 0.564) out-performed models that indicated spatial displacement (ψ^ER/WB^ < ψ^ER/wb^; AICw = 0.116). Furthermore, the average SIF across the model set supports a scenario of independent occupancy (SIF = 1.003 ± 0.114). Regarding our second hypothesis of wild boar’s presence negatively influencing rabbit’s detection probability, the evidences supported species interaction (pER <rER/WB; AICw= 0.247). For these models, rabbit’s detection probability was higher when wild boar was present and averaging across the model set further supported this interaction with estimates of pER = 0.383 ± 0.068 and rER/WB = 0.489 ± 0.087 (Fig. 2).

SIF estimates for models considering rabbit’s occupancy conditional upon wild boar’s presence exhibited opposite signs depending on the parameterization of rabbit’s detection probability. When rabbit’s occupancy was conditional upon wild boar’s presence, but detection was independent (AICw = 0.322), the models suggested slight co-occurrence (SIF=1.067 ± 0.276). Models that assumed simultaneously conditional occupancy and detection for rabbit (AICw=0.116), estimated an avoidance pattern instead (SIF=0.845 ± 0.217) (Table 3).

In terms of covariate effect, model average of the beta coefficients indicated a positive effect of shrub cover on European rabbit’s detection probability (β = 0.158 ± 0.757), but the distance to cultivated areas had a negative effect on species occupancy (β = −0.362 ± 0.484). For wild boar, detection probability was negatively influenced by litter cover (β = −0.413 ± 0.43) and the proportion of pine stands had a negative influence on species occupancy (β = −0.535 ± 0.639). However, the large SEs hindered the accurate assessment of the predictors’ influence (see Supplementary Table S6), although they had a well-supported effect in the preliminary stage.

## Discussion

Our study explored the different pathways of European rabbit and wild boar interaction in an agroforestry farmstead. This fine-scale analysis demonstrated the importance of considering interdependencies between conditional detection and occupancy, an issue often overlooked when unveiling ecological patterns.

The results from occupancy modelling supported our previous knowledge of the study area^43^: European rabbit had a low occupancy while the wild boar was widespread. Furthermore, the evidences indicated an independent occurrence pattern of European rabbit and wild boar in the study area, which did not support our first hypothesis. Wild boar’s wide distribution most likely hindered predictions regarding species co-occurrence, since sites were wild boar was absent were too scarce to draw inferences. Also, the small study area and depleted rabbit population further limited the assessment of species co-occurrence.

Nonetheless, there was some support for interaction in species detection, which indicated that wild boar’s presence positively influenced rabbit’s detection probability. The model averaged estimates showed that rabbit detection was higher when the wild boar was present, a result that supported our second hypothesis but contrariwise to what was expected. This could have resulted from the combined effect of wild boar’s influence on rabbit's behaviour and surveying success. Wild boar’s rooting creates open areas which rabbits could have used more intensively for marking^61^ and this would have increased detectability. Also, these open areas may increase observer’s visibility (and thus detection) in areas of dense vegetation. This effect could be greater in low density populations where scattered pellets are more common than big latrines^48^. However, further research into the factors mediating such an interaction, either through behavioural or methodological studies, are necessary.

Assuming this positive effect on rabbit’s detection, we further analysed how this parameter influenced interpretations of species co-occurrence. For models where occupancy was conditioned but detection was not, the pattern was of slight co-occurrence; but for models where both occupancy and detection were conditioned the resulting pattern was of spatial displacement. Although these interaction patterns did not have a strong support, this suggests that failing to account for an interaction in species detection probability could lead to erroneous interpretations of wild boar’s influence on rabbit’s occupancy pattern. Ultimately, if species interaction for occupancy and detection probabilities have opposite signs (positive effect on detection, and negative on occupancy), not accounting for wild boar’s positive effect on detection could lead to interpretations of independent occurrence or even co-occurrence. Furthermore, these interdependencies could contribute to mask similar spatial interactions between species’ pairs^62^, especially for species that affect the habitat’s physical structure^63,64^ and hence potentially influence detectability indirectly. Several authors have demonstrated the importance of considering imperfect detections^65^, but only a few have verified an interaction in detection patterns^4,17,66^.

When modelling single-species occupancy and detection most of the covariates included in top-ranked models were well-supported. The positive effect of percentage of shrub cover on rabbit’s detection can potentially reflect increased intensity of fine-scale habitat use in more vegetated patches^50^, increasing detectability. Such effect may surpass an anticipated negative influence by reduced visibility of signs of presence. For occupancy, an association to cultivated areas is well documented, since this is an important food resource for rabbit^67^, especially in Mediterranean ecosystems, characterized by strong fluctuations in annual primary production^68^. Wild boar’s detection probability was lower when litter cover was high, probably because during the sampling period, dead leaf cover, especially in Montado, was abundant and could have reduced visibility of signs of presence (i.e. footprints and droppings). A lower occupancy of wild boar in sites with higher proportion of pine stands could be due to the low shrub cover which reduced suitability as a refuge. However, the influence of the landscape variables seems to be unclear, since in the final two-species model framework none of the covariates included had a well-supported effect. The most likely explanation for this outcome is that the covariates were required because the influence of the other species presence on detection and occupancy was being ignored. Once it was accounted for these covariates’ significance was no longer supported.

The small-scale of our study and the current population scenario of European rabbit and wild boar in the area, limited our assessment of a co-occurrence pattern. Therefore, we recommend that field sampling design considers a landscape approach to capture a gradient of wild ungulates’ and European rabbits’ densities. Also, the use of species abundance instead of binary data could make this pattern more evident at larger scales. Nonetheless, our study was a preliminary approach and resorting to occupancy modelling allowed us to evaluate potential interactions in detection and occupancy, but also the interdependencies between them. The above-mentioned limitations also hindered a definitive assessment of wild boar’s effect on rabbit’s detection. However, if the suggested positive effect on detection probability is confirmed, then failing to account for it could falsely indicate a co-occurrence pattern. Understanding the reasons behind this interaction requires more in-depth knowledge into European rabbit’s fine-scale habitat use. Besides, even if assessing co-occurrence is not of interest *per se*, this means one could overestimate rabbit’s presence in areas of wild boar presence.

Therefore, despite the limitations, this complexity of detection and occupancy interdependencies should be acknowledged in future research. Especially, for other wild and domestic ungulates with similar foraging behaviour that creates open areas, potentially influencing rabbit’s detection. Furthermore, questions may also arise for any other setting in which the influence of a species promotes the detection of a second one, leading to potential bias if ignored. We reinforce the urgency of research on the potential threat by ungulates and hope that our results and recommendations will better guide future research on this topic. This is particularly relevant since the European rabbit is central to many species conservation plans in Mediterranean ecosystems^69^ and has just recently been reclassified as “Endangered” worldwide by the IUCN^40^.

## Supplementary information

Supplementary Information.

## Data Availability

The datasets generated during and/or analysed during the current study are available from the corresponding author on reasonable request.
