# Supplementary material for: Accounting for detection unveils the intricacy of wild boar and rabbit co-occurrence patterns in a Mediterranean landscape
Source: Sci Rep. 2020 Apr 20;10:6651. doi: 10.1038/s41598-020-63492-9 (PMC7170872; doi:10.1038/s41598-020-63492-9)
Supplement: Supplementary file 1 — Supplementary Information. [file 41598_2020_63492_MOESM1_ESM.pdf]

Supplementary Material

Accounting for detection unveils the intricacy of wild boar and rabbit co-occurrence patterns in a Mediterranean landscape

Ana Luísa Barros* [a], Gonçalo Curveira-Santos [a], Tiago André Marques [b,c], Margarida Santos-Reis [a]

[a] cE3c – Centre for Ecology, Evolution and Environmental Changes, Faculdade de Ciências da Universidade de Lisboa, Ed. C2, Campo Grande, 1749-016 Lisbon, Portugal

[b] Centre for Research into Ecological and Environmental Modelling, The Observatory, University of St Andrews, St Andrews, KY16 9LZ, Scotland;

[c] Centro de Estatística e Aplicações, Departamento de Biologia Ambiental, Faculdade de Ciências, Universidade de Lisboa, 1749-016 Lisbon, Portugal

*Corresponding author: albarros@fc.ul.pt; 00351910464091

	SH	S	HH	H	L	B
**SH**	1					
**S**	0.855*	1				
**HH**	-0.122	-0.134	1			
**H**	-0.805*	-0.757*	0.383	1		
**L**	0.01	0.079	-0.435	-0.894*	1	
**B**	-0.229	-0.223	-0.081	-0.17	0.153	1

Supplementary Table S1 – Results of pairwise Spearman’s correlation where * indicates a strong correlation (>0.7) between detection covariates. Covariate abbreviations are shrub cover (S), herbaceous cover (H), litter cover (L), shrub height (SH), herbaceous height (HH) and bare ground (B).

	Model	K	AICc	ΔAICc	AICw
**European rabbit**	Ψ(Cult),p(S)	4	196.11	0	0.234
	Ψ(.),p(S)	3	197.02	0.91	0.149
	Ψ(Pine),p(S)	4	197.83	1.73	0.099
	Ψ(Dense),p(S)	4	198.32	2.22	0.077
	Ψ(Cult + Art),p(S)	5	198.4	2.3	0.074
	Ψ(Cattle),p(S)	4	198.64	2.53	0.066
	Ψ(Div),p(S)	4	199.1	2.99	0.052
	Ψ(Sparse),p(S)	4	199.13	3.02	0.052
	Ψ(Art),p(S)	4	199.13	3.02	0.052
	Ψ(Absent),p(S)	4	199.25	3.14	0.049
	Ψ(Mont),p(S)	4	199.25	3.15	0.049
	Ψ(Mont + Sparse + Cult),p(S)	6	200.55	4.44	0.025
	Ψ(Div + Cattle),p(S)	5	200.82	4.71	0.022
**wild boar**	Ψ(Pine),p(L)	4	284.89	0	0.254
	Ψ(.),p(L)	3	285.99	1.1	0.146
	Ψ(Rip),p(L)	4	286.96	2.07	0.09
	Ψ(Cattle),p(L)	4	287.08	2.18	0.085
	Ψ(Mont + Pine),p(L)	5	287.17	2.28	0.081
	Ψ(Mont),p(L)	4	287.18	2.28	0.081
	Ψ(Mont + Rip),p(L)	4	287.48	2.59	0.07
	Ψ(Div),p(L)	4	288.2	3.31	0.049
	Ψ(Absent),p(L)	4	288.21	3.31	0.048
	Ψ(Cult),p(L)	4	288.22	3.32	0.048
	Ψ(Sparse),p(L)	4	288.23	3.34	0.048

Supplementary Table S2 – Ranking of the single-season single-species occupancy models for European rabbit and wild boar, using the detection covariate from the best model obtained in the previous analysis. For covariates' codes see table 2. Goodness-of-fit tests suggested an adequate fit to the data (European rabbit: p-value = 0.068, c-hat = 1.96; Wild boar: p-value = 0.66, c-hat = 0.74).

		European rabbit			wild boar		
	Covariates	*β coefficient ± SE*	*CI_{inf}*	*CI_{sup}*	*β coefficient ± SE*	*CI_{inf}*	*CI_{sup}*
Detection	L				-0.42 ± 0.2*	-0.74	-0.09
	S	0.47 ± 0.25*	0.06	0.87			
Occupancy	Pine	0.32 ± 0.28	-0.13	0.77	-0.83 ± 0.41*	-1.5	-0.16
	Cult	-0.55 ± 0.33*	-1.09	-0.01			

Supplementary Table S3– Model averaged beta coefficient estimates, standard error (SE) and 90% confidence interval (CI) for covariates included in the well supported single-species single-season models for European rabbit and wild boar respectively. * indicates a well-supported effect.

	Model	*K*	*AICc*	*ΔAICc*	*AICcw*
**European rabbit**	Ψ(.),p(S)	3	196.67	0	0.384
	Ψ(.),p(.)	2	198.18	1.51	0.18
	Ψ(.),p(HH)	3	198.25	1.58	0.174
	Ψ(.),p(B)	3	198.97	2.3	0.121
	Ψ(.),p(L)	3	199.95	3.28	0.074
	Ψ(.),p(JD)	3	200.18	3.51	0.066
**wild boar**	Ψ(.),p(L)	3	285.65	0	0.839
	Ψ(.),p(.)	2	290.93	5.28	0.06
	Ψ(.),p(HH)	3	292.44	6.79	0.028
	Ψ(.),p(B)	3	292.47	6.82	0.028
	Ψ(.),p(S)	3	292.81	7.16	0.023
	Ψ(.),p(JD)	3	292.93	7.28	0.022

Supplementary Table S4– Ranking of the single-season single-species models for detection probability of European rabbit and wild boar. For covariates' codes see Table 2. Goodness-of-fit tests suggested an adequate fit to the data (European rabbit: p-value = 0.068, c-hat = 1.96; Wild boar: p-value = 0.66, c-hat = 0.74).

Hypothesis	*Model*	*K*	*AIC*	*ΔAIC*	*AICw*	*SIF*
Ψ(un), p(un)	ΨWB(Pine) ΨER(Cult) ρWB(L) ρER(S)	8	479.82	0	0.165	1
Ψ(cond), p(un)	ΨWB(.) ΨER/WB(Cult) ΨER/wb(.) ρWB(L) ρER(S)	8	480.81	0.99	0.101	>1
Ψ(un), p(un)	ΨWB(Pine) ΨER(.) ρWB(L) ρER(S)	7	480.97	1.15	0.093	1
Ψ(cond), p(un)	ΨWB(Pine) ΨER/WB(Cult) ΨER/wb(.) ρWB(L) ρER(S)	9	480.98	1.16	0.093	>1
Ψ(un), p(un)	ΨWB(.) ΨER(Cult) ρWB(L) ρER(S)	7	481.16	1.34	0.085	1
Ψ(un), p(cond)	ΨWB(Pine) ΨER(Cult) ρWB(L) ρER(S) rER/WB=rER/wb(.)	9	481.67	1.85	0.066	1
Ψ(cond), p(cond)	ΨWB(Pine) ΨER/WB(Cult) ΨER/wb(.) ρWB(L) ρER(S) rER/WB=rER/wb(.)	10	481.69	1.87	0.065	<1
Ψ(un), p(cond)	ΨWB(Pine) ΨER(.) ρWB(L) ρER(S) rER/WB=rER/wb(.)	8	482.12	2.3	0.052	1
Ψ(cond), p(un)	ΨWB(Pine) ΨER/WB(.) ΨER/wb(.) ρWB(L) ρER(S)	8	482.16	2.34	0.051	>1
Ψ(un), p(un)	ΨWB(.) ΨER(.) ρWB(L) ρER(S)	6	482.31	2.49	0.048	1
Ψ(cond), p(un)	ΨWB(.) ΨER/WB(.) ΨER/wb(.) ρWB(L) ρER(S)	7	482.42	2.6	0.045	>1
Ψ(cond), p(cond)	ΨWB(Pine) ΨER/WB(.) ΨER/wb(.) ρWB(L) ρER(S) rER/WB=rER/wb(.)	9	482.66	2.84	0.04	<1
Ψ(cond), p(cond)	ΨWB(.) ΨER/WB(Cult) ΨER/wb(.) ρWB(L) ρER(S) rER/WB=rER/wb(.)	9	482.86	3.04	0.036	<1
Ψ(un), p(cond)	ΨWB(.) ΨER(Cult) ρWB(L) ρER(S) rER/WB=rER/wb(.)	8	483.24	3.42	0.03	1
Ψ(un), p(cond)	ΨWB(.) ΨER(.) ρWB(L) ρER(S) rER/WB=rER/wb(.)	7	483.98	4.16	0.021	1
Ψ(cond), p(cond)	ΨWB(.) ΨER/WB(.) ΨER/wb(.) ρWB(L) ρER(S) rER/WB=rER/wb(.)	8	485.17	5.35	0.011	<1

Supplementary Table S5– Ranking of the single-season two-species occupancy models following the parameterization of Richmond et al., 2010. In the hypothesis column, “un” stands for unconditional and “cond” for conditional occupancy or detection probability. For the description of model parameters see Table 1 and for covariates’ codes see Table 2.

		European rabbit			wild boar		
	Covariates	*β coefficient ± SE*	CI_{inf}	CI_{sup}	*β coefficient ± SE*	CI_{inf}	CI_{sup}
Detection	L				-0.413 ± 0.43	-1.12	0.294
	S	0.158 ± 0.757	-1.087	1.402			
Occupancy	Pine				-0.535 ± 0.639	-1.585	0.516
	Cult	-0.362 ± 0.484	-1.158	0.435			

Supplementary Table S6– Model averaged beta coefficient estimates, standard error (SE) and 90% confidence interval (CI) for covariates included in the two-species single-season models for European rabbit and wild boar interaction. * indicates well-supported covariates.
